# Immunological effects of nilotinib prophylaxis after allogeneic stem cell transplantation in patients with advanced chronic myeloid leukemia or philadelphia chromosome-positive acute lymphoblastic leukemia

**DOI:** 10.18632/oncotarget.13439

**Published:** 2016-11-18

**Authors:** Nira Varda-Bloom, Ivetta Danylesko, Roni Shouval, Shiran Eldror, Atar Lev, Jacqueline Davidson, Esther Rosenthal, Yulia Volchek, Noga Shem-Tov, Ronit Yerushalmi, Avichai Shimoni, Raz Somech, Arnon Nagler

**Affiliations:** ^1^ Sheba Medical Center, Ramat-Gan, Israel; ^2^ Sackler Faculty of Medicine, Tel-Aviv University, Israel; ^3^ Dr. Pinchas Bornstein Talpiot Medical Leadership Program, Sheba Medical Center, Israel; ^4^ Bar-Ilan University, Ramat Gan, Israel; ^5^ Pediatric Immunology Service, Jeffrey Modell Foundation, USA; ^6^ Edmond and Lily Safra Children's Hospital, Israel

**Keywords:** nilotinib, stem cell transplantation, immune reconstitution, mitogens, thymic activity

## Abstract

Allogeneic stem cell transplantation remains the standard treatment for resistant advanced chronic myeloid leukemia and Philadelphia chromosome–positive acute lymphoblastic leukemia. Relapse is the major cause of treatment failure in both diseases. Post-allo-SCT administration of TKIs could potentially reduce relapse rates, but concerns regarding their effect on immune reconstitution have been raised. We aimed to assess immune functions of 12 advanced CML and Ph+ ALL patients who received post-allo-SCT nilotinib. Lymphocyte subpopulations and their functional activities including T-cell response to mitogens, NK cytotoxic activity and thymic function, determined by quantification of the T cell receptor (TCR) excision circles (TREC) and TCR repertoire, were evaluated at several time points, including pre-nilotib-post-allo-SCT, and up to 365 days on nilotinib treatment. NK cells were the first to recover post allo-SCT. Concomitant to nilotinib administration, total lymphocyte counts and subpopulations gradually increased. CD8 T cells were rapidly reconstituted and continued to increase until day 180 post SCT, while CD4 T cells counts were low until 180−270 days post nilotinib treatment. T-cell response to mitogenic stimulation was not inhibited by nilotinib administration. Thymic activity, measured by TREC copies and surface membrane expression of 24 different TCR Vβ families, was evident in all patients at the end of follow-up after allo-SCT and nilotinib treatment. Finally, nilotinib did not inhibit NK cytotoxic activity. In conclusion, administration of nilotinib post allo-SCT, in attempt to reduce relapse rates or progression of Ph+ ALL and CML, did not jeopardize immune reconstitution or function following transplantation.

## INTRODUCTION

Tyrosine kinase inhibitors (TKIs) are the standard front line targeted therapy in CML and an integral part of combination chemotherapy for Philadelphia-positive acute lymphoblastic leukemia (Ph+ ALL). TKIs have led to improvement in rates of complete remission and overall outcomes in both CML as well as Ph+ ALL [1, 2]. The role of allogeneic stem cell transplantation (allo-SCT) in CML is reserved for patients who fail or are intolerant to TKI therapy, or for those with advanced accelerated and blast phase disease [3]. Transplantation remains the standard of care for Ph+ ALL [2].

Relapse rates after allo-SCT are as high as 30–40% and 30–60% for both advanced phase CML and Ph+ ALL, respectively [4, 5]. Therapeutic strategies to prevent or treat relapse after allo-SCT include withdrawal of immunosuppression and donor lymphocyte infusions (DLI) [6, 7]. Given their value in treating relapsed CML or Ph+ ALL [8–15], TKIs were also studied as part of a preemptive or prophylactic approach post allo-SCT. Several groups have evaluated imatinib for the prevention of relapse in high-risk CML and Ph+ALL patients with encouraging results [16–20]. Second generation TKIs, including nilotinib, may be more efficient in preventing and treating relapse of advanced CML and Ph+ ALL [7, 21]. However, data on their use in the post allo-SCT setting are limited [7, 22, 23].

The success of allo-SCT is dependent on both hematopoietic stem cells' engraftment and immune reconstitution. Immune reconstitution is a complex process requiring functional hematopoietic stem cells in the bone marrow niche and adequate thymic function [24]. Without full reconstitution of the immune system following allo-SCT, patients are more susceptible to life threatening viral and fungal infections. Furthermore, they are also prone to tumor relapse, as graft-versus-leukemia (GVL) depends on a functional immune system [25, 26]. CML is particularly sensitive to allogeneic immune reactivity; there is a dominant GVL effect induced by DLI in treating disease relapse, and improved leukemia-free survival in patients with graft-versus-host disease (GvHD) [27, 28].

The interaction between post-allo-SCT TKI administration and immune reconstitution is of major importance, given the critical role of the latter in the course following transplantation, as detailed above. In several *in-vitro* studies, inhibition of the innate immune cells activation as well as T-cell proliferation and function were noted [24, 29–32]. However, others have reported that patients treated with TKI have near-normal levels of immunological parameters and *ex-vivo* response to various cytokine stimuli [27]. Thus, the literature is inconsistent regarding the effects of TKIs on the immune system in the post-allo-SCT setting.

We recently reported the clinical outcomes of a phase 1/2 study in CML and Ph+ ALL patients treated with nilotinb after allogeneic SCT. Nilotininb was safe and partially effective for the prevention of relapse after allo-SCT [23]. In the current study, we further explored nilotinib effect on immune reconstitution post allo-SCT. Our aim was to quantitatively characterize immune subpopulations and evaluate their function including T-cell response to mitogens, NK cytotoxic activity, and T-cell repertoire and thymic activity (TREC) at designated time points up to 1 year after transplantation while on nilotinib therapy.

## RESULTS

### Total cell numbers

**Figure 1:**
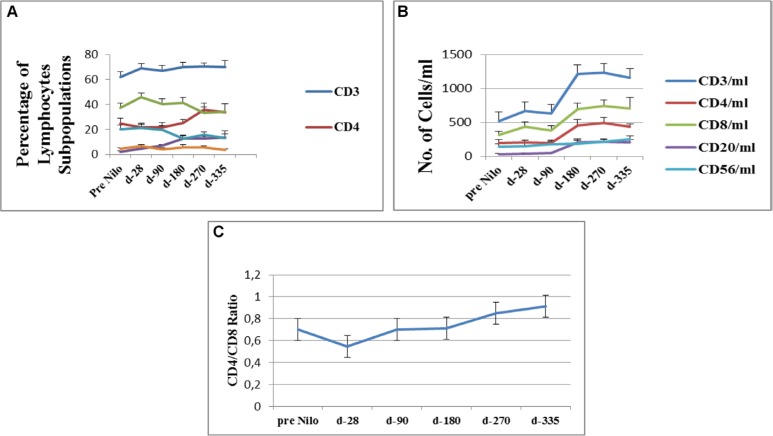
Flow cytometry analysis of lymphocytes subpopulations (**A**) Percentage of cells expressing specific lymphocytes surface markers (CD3, CD4,CD8, CD20 and CD56). (**B**) Average concentration of lymphocytes subpopulations, calculated from their percentage on gated CD45pos cells. (**C**) CD4/CD8 ratio calculated from their concentration at each study time point. CD - cluster of differentiation.

**Figure 2:**
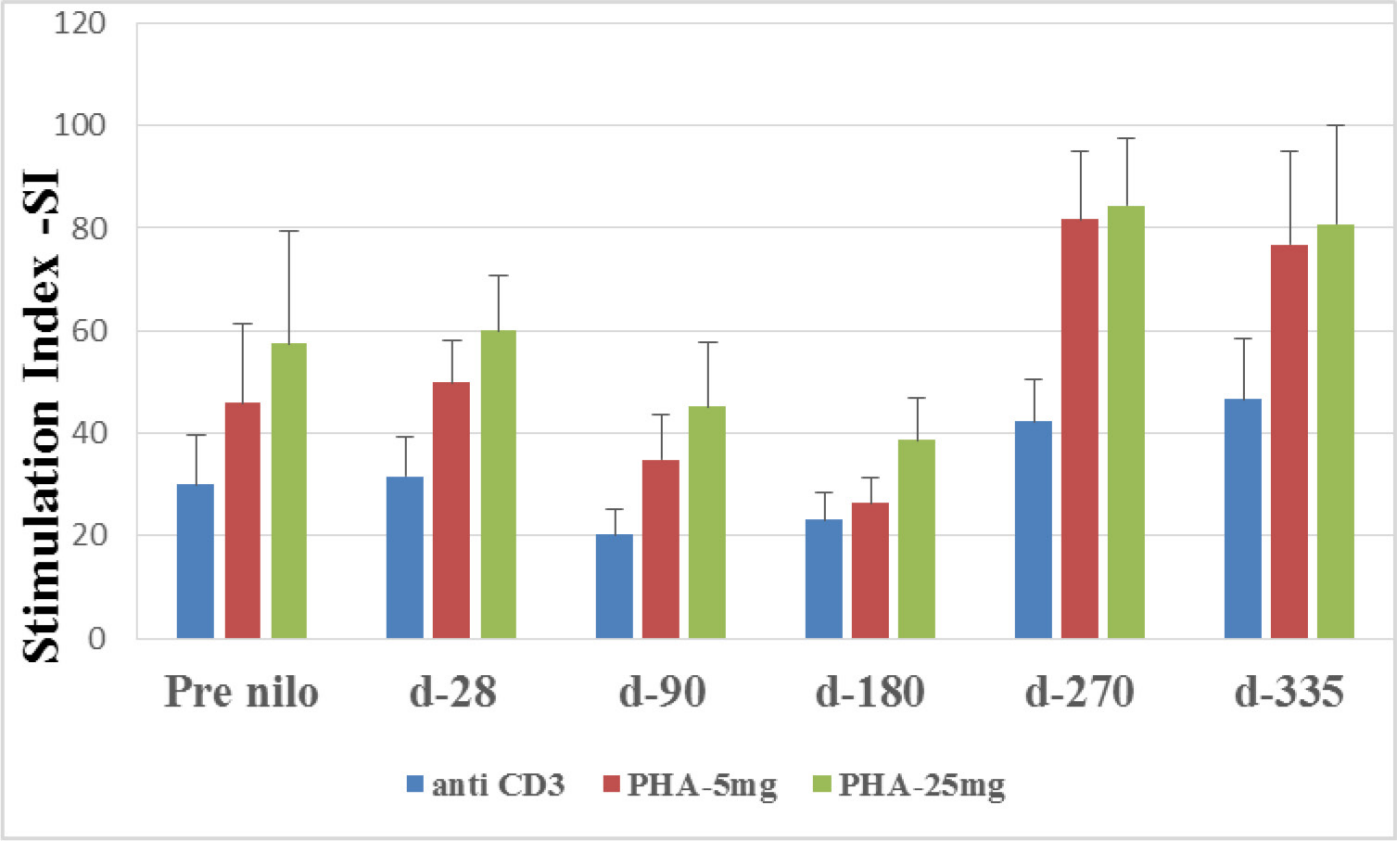
Stimulation Index (SI) of T lymphocytes T lymphocytes function was tested by their activation abilities to different mytogene at different time points after allo-SCT. SI was calculated as the ratio of (3H) Thymidine uptake of stimulated cells divided by (3H) Thymidine uptake of non-stimulated cells. SI- Stimulation Index, CD- cluster of differentiation, PHA- phytohaemagglutinine.

The relation between total white blood cells (WBC) and lymphocytes was analyzed directly from complete blood counts (Figure 1 and Table 1). Mean (± standard error) WBC at day 28 of nilotninb treatment (4014 ± 398 cells/ml) was similar to that measured post allo-SCT and before nilotinib treatment (4137 ± 600 cells/ml), whereas a significant increment of WBC was observed at day 90 of nilotinib treatment (5887 ± 771 cells/ml, *p* = 0.04). WBC counts continued to increase thereafter, with a mean of 9250 ± 904 cells/ml on day 335 (Figure 2). When compared to their level at day 28 of nilotinib administration, an increase in total lymphocytes was first noted at day 180 (1693.7 ± 166.6 vs. 942.8 ± 120.6 cells/ml, *p* < 0.001, respectively). Lymphocyte counts were maintained up to day 335 post nilotinib administration (Table 1).

**Table 1:** Immune reconstitution after allo-SCT during nilotinib treatment

	Pre nilotinib	Day 28	Day 90	Day 180	Day 270	Day 335
**Total WBC/ml**	4.138 ± 600.5	4.014 ± 3.98	5.877 ± 771	7.081 ± 618.4	7.810 ± 608	9.250 ± 905
**Total Lym/ml**	820 ± 148.5	943 ± 120.6	924.3 ± 102.6	1693.7 ± 167	1,754 ± 109.4	1,647 ± 147
**CD3/ml** **Percentage**	523.7 ± 78 62% ± 4.3	665.3 ± 89.8 69% ± 3.8	633.7 ± 87.2 67% ± 4.1	1213.3 ± 146 69.9% ± 3.9	1233.3 ± 71.2 70.3% ± 2.6	1160.8 ± 153 70% ± 5.4
**CD4/ml** **Percentage**	199.6 ± 46.8 24.8 ± 4.03	202.8 ± 37.7 21.7 ± 3.2	197.3 ± 38.7 21.9 ± 3.5	457.1 ± 87.5 25.2 ± 3	490.7 ± 77.1 35.8 ± 5.3	434.5 ± 45 33.8 ± 6.5
**CD8/ml** **Percentage**	318.1 ± 52 37.1 ± 3.7	437.1 ± 67.8 45.9 ± 3.3	385.3 ± 69.3 40 ± 4.7	696.8 ± 88.1 41.1 ± 4.7	738.1 ± 93.0 33.1 ± 4.7	704.5 ± 158.0 34.5 ± 6.3
**CD20/ml** **Percentage**	30.0 ± 14 2.1 ± 0.6	39.1 ± 11.9 4.7 ± 1.4	45.7 ± 7.3 7 ± 0.86	210.3 ± 44.4 28.3 ± 16.4	218.3 ± 30.1 12.6 ± 1.8	208.0 ± 41.1 13.7 ± 5.5
**CD56/ml** **Percentage**	145 ± 35.3 20.4 ± 3.1	146.9 ± 41.5 21 ± 2.8	175.2 ± 41.8 19.8 ± 3.1	189.0 ± 48.8 12.5 ± 2.3	215 ± 44.9 15.8 ± 2.3	255.1 ± 49.2 13 ± 3.4

Total WBC and total lymphocytes were tested by CBC counts. T cells subpopulation counts were analyzed by Flow Cytometry. Calculations of cells percent and concentrations were performed on gated CD45^pos^ cells.

Abbreviations: WBC - white blood cells, CD - cluster of differentiation.

### Lymphocyte subpopulations

#### CD3^pos^ T-cells

Lymphocyte subpopulations were identified from a gated CD45^pos^ cell population. Starting after allo-SCT pre-nilotinib administration a constant high percentage of CD3^pos^ T lymphocytes was observed (62% ± 4.3) (Figure 1A). CD3^pos^ cells numbers significantly increased at day 180 after nilotinb administration (mean 1213.3 ± 146 × 10^6^/ml; *p* < 0.001) compared to their numbers at day 28 and at day 90 (665.3 ± 89.8 × 10^6^/ml and 633 ± 87 × 10^6^/ml, respectively). CD3^pos^ T-cell counts were maintained at day 270 and up to the last assessment at day 335 (Figure 1B, Table 1).

#### CD4^pos^ T-cells

The percentage of CD4^pos^ cells began to increase at day 270 of nilotinib administration (35.8 ± 5.3%; *p* = 0.06) compared to values measured pre-nilotinb administration. (Figure 1A, Table 1). CD4^pos^ cell counts significantly increased at day 180 (457.1 ± 87.5 × 10^6^/ml; *p* = 0.01 compared to their values at day 28 (202.8 ± 37.7 × 10^6^/ml). Counts remained stable at day 270 (490.7 ± 77.1 × 10^6^/ml) and at day 335 (434.5 ± 44.9 × 10^6^/ml), respectively (Figure 1B).

#### CD8^pos^ T-cells

The percentage of CD8^pos^ cells remained stable from post-allo-SCT-pre-nilotinib until the last evaluation at day 335 (Figure 1A). An increase in CD8^pos^ cells was observed after 180 days of nilotinib treatment (696.8 ± 88 × 10^6^/ml), compared to their measurement at the post-allo-SCT-pre-nilotinib time point (318.1 ± 52 × 10^6^/ml; *p* = 0.001); (Figure 1B, Table 1). These results effect the CD4/CD8 ratio, which was calculated to evaluate immune system activity potential.

#### CD4/CD8 ratio

The CD4/CD8 ratio consistently increased from day 28 of nilotinib administration (0.55 ± 0.18); 0.7 ± 0.11, 0.85 ± 0.17, and 0.91 ± 0.21 at days 180, 270, and 335, respectively (Figure 1C).

#### CD20^pos^ B-Cells

The percent of CD20^*pos*^ cells out of total lymphocytes significantly increased at day 180 (12.9 ± 2.6%; *p* > 0.001), with no difference at day 270 (12.6 ± 1.8%) and on day 335 (13.7 ± 5.5%), compared to CD20^*pos*^ percent found post allo-SCT-pre-nilotinib administration. CD20^*pos*^ counts increased at day 180 (210.3 ± 44.4 × 10^6^/ml; *p* = 0.002) compared to CD20^*pos*^ counts found post allo-SCT-pre-nilotinib administration and remained stable up to day 335 (Figure 1A–1B, Table 1).

#### NK cells (CD56^pos^, CD3^negative^)

The percent of CD56^pos^ cells out of the total lymphocyte population was stable from post allo-SCT-pre-nilotinib (20.4 ± 3.14%) up to day 90 post nilotinib administration, following a significant reduction at day 180 (12.5 ± 2.2%; *P* < 0.04). Counts of Natural Killer cells increased insignificantly from day 90 (*P* = 0.32) to day 335 (*p* = 0.18) post nilotinib administration, compared to their values pre-nilotinib and at day 28 (146.9 ± 41.5 × 10^6^/ml) (Figure 1A–1B, Table 1).

### Mitogenic responses

Lymphocyte function was assessed by their ability to proliferate in response to stimulation by different mitogens (see material and methods). The average stimulation index (SI) values obtained after anti-CD3 (αCD3) cross-linking decreased insignificantly at day 90 (20.1 ± 5.2) compared to the values obtained pre-nilotinib (30 ± 9.6; *p* = 0.1) and at day 28 post nilotinib administration (31.6 ± 7.5; *p* = 0.1), respectively (Figure 2). A decline in the pick stimulation index was observed at day 180 (23 ± 5.4), while a modest insignificant increment was measured at days 270 and 335 post nilotinib administration (42.3 ± 8, *p* = 0.1; 46.7 ± 11.7, *p* = 0.1; respectively) (Figure 2).

Similar results were observed in response to PHA at 5 mg and 25 mg. Pick low stimulation index was observed at day 180 (26.3 ± 5.2; and 38.6 ± 8.3; respectively), increased at day 270 (81 ± 13.3; *p* = 0.02 and 84.5 ± 13.1; *p* = 0.03, respectively) and remained high at day 335 (76.8 ± 18.3; *p* = 0.05 and 80.7 ± 19.4; *p* = 0.09) (Figure 2).

### Cytotoxic activity

**Figure 3:**
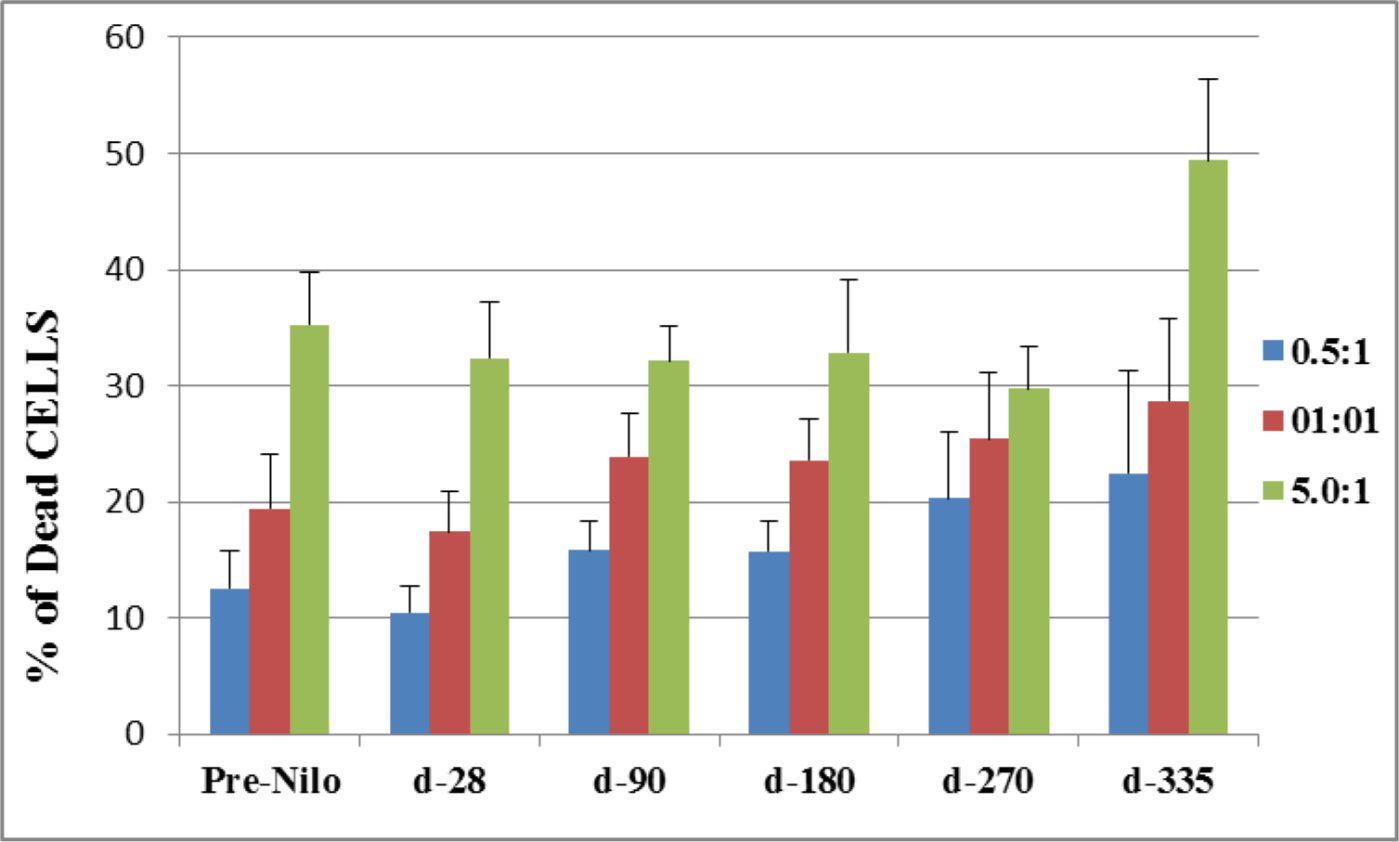
Cytotoxic activity of NK effector cells against K562 cell line as target cells The cytotoxic activity of NK cells is presented as the percent of dead target cells at d-28, 90, 270 and 335 post nilotinib administration, after co-culture at different E:T ratios. NK cells were obtained from peripheral blood samples of post SCT patients treated with nilotinib. NK–natural killers, E-effector, T- target.

Natural killer cells function was evaluated by their cytotoxic activity against K562 cells. Figure 3 shows the percentage of dead target cells (K562 cell line) following co-culture with patients NK cells (in different E:T ratios). At all time points, increased E:T ratios, resulted in correlated higher percentage of specific killing, and maximal effect was found at 5:1 E:T ratio. Moreover, a similar percent of target cells death were determined up to day 270 post nilotinib treatment, compared to target cell death prior to nilotinib administration. Specific killing was significantly increased at day 335 post nilotinib administration (49.4 ± 7; *p* = 0.05), compared to values at day 270 (29.7 ± 3.7) (Figure 3).

### Thymic activity

In order to assess thymic activity, TREC quantification and analysis of the TCR repertoire were performed. Before treatment and despite immunosuppression from previous interventions, the average pre-nilotinb TREC levels were 81.8 ± 108 copies per 0.5 ug DNA. At the end of follow-up, after patients received allo-SCT and nilotinib treatment, TRECs levels increased (142.8 ± 197.4 copies per 0.5 ug DNA). However, this trend did not reach statistical significance. (Table 2) Similarly, the surface membrane expression of 24 different TCR Vβ (TRBV) post-treatment was not different from baseline expression. At the end of follow up, patients had normal expression of 15.1 ± 5.5 receptors (of the 24 that were examined), 2.5 ± 2.2 receptors with clonal expression and 6.4 ± 4.3 receptors with reduced expression. These numbers were similar to the expression of the TCR Vβ before allo-SCT and nilotinib treatment (normal receptors 15.3 ± 5.6, clonal receptors 2.9 ± 3 and reduced receptors 5.8 ± 4.5, respectively).

**Table 2:** TREC content and TCR repertoire

	Pre Nilotinib treatment	Post Nilotinib treatment
TREC content (copies/0.5 ug DNA)	81.8 ± 108	142.8 ± 197.4
TCR repertoire – normal expression	15.3 ± 5.6	15.1 ± 5.5
TCR repertoire – clonal expression	2.9 ± 3	2.5 ± 2.2
TCR repertoire – reduced expression	5.8 ± 4.5	6.4 ± 4.3

TREC content and surface membrane expression of 24 different T cell receptor Vβ families in patients' samples collected before and at the last nilotinib treatment last time point. TCR repertoire for each patient was calculated as percentage of normal expression, clonal expression and reduced expression.

Abbreviations: TREC - TCR excision circles, TCR **-** T cell receptor.

## DISCUSSION

We have recently demonstrated the feasibility, safety, and efficacy of nilotinib, a second-generation TKI, as maintenance therapy after allogeneic SCT in patients with advanced CML and Ph+ ALL [23]. In the current study, we show that nilotinb administration up to one year following allogeneic SCT does not jeopardize immune reconstitution and function in the recipients.

TKIs are increasingly used as a complementary therapy after SCT. Several phase 2 studies explored use of imatinib treatment after SCT and have shown promising outcomes [16–20, 33]. Recently, Pfeifer et al., published a randomized trial studying the role of TKIs in the post SCT in patients with Ph+ ALL, comparing prophylactic imatinib and preemptive imatinib therapy, triggered by the detection of minimal residual disease (MRD) [19]. Post-transplant imatinib resulted in low relapse rates and excellent outcomes that were equivalent in both arms [19].

Findings from pre-clinical studies on the effect of imatinib on the immune response are conflicting, ranging from impaired antigen-specific T-cell responses [34–36] to reversal of T-cell tolerance and potentiation of antitumor immune responses [37, 38]. Limited *in vitro* data that are available with second-generation TKIs, including nilotinib, showed impaired antigen-specific T-cells, NK cell response, and B-cell activation [29, 31, 39–42]. However, recent clinical studies reported that dasatinib enhances NK and NK/T-cell proliferation and activation [43].

The speed of immune recovery after SCT is of central importance for overcoming complications. A rapid immune reconstitution post SCT protects the transplanted patients from severe infections and other transplant related complications as well as relapse [44–47]. The aim of our study was therefore to follow the effect of post-transplantation nilotinib administration on immune reconstitution after allogeneic SCT. Quantitative and functional aspects of the recipients' immune system were studied during nilotinib administration and throughout the post-transplantation course (from day 28 through day 335). The former was evaluated through characterization of lymphocyte sub-populations, while the latter was investigated by studying T-cell proliferation in response to mitogenic induction and killing assay mediated by NK cells. In addition, thymic function was determined by TREC and TCR repertoire. TREC are by-products of V(D)J recombination at the TCR V/β locus and their presence indicates de-novo lymphopoiesis. This was found to be an excellent predictor for T cell immune reconstitution after SCT [48]. A normal TCR repertoire reflects ability of T-cells to produce a required diversity.

Following SCT there is severe depletion of all hematopoietic cells of the immune system, especially lymphocytes. Previous studies showed that innate immunity recovers within weeks after allogeneic SCT, whereas adaptive immunity (B- and T-cells) recovers within months to years [49, 50]. NK cells (CD16- CD56+), as part of innate immunity, recover both numerically and functionally within the first few weeks following SCT [51]. Our findings confirm the rapid recovery of NK cells, reaching the normal adult level even before nilotinib treatment. Moreover, their level remained stable during nilotinib treatment.

Previous studies indicated that the recovery of lymphocytes and their subpopulation is a major predictor of transplantation success [52]. In this context, our finding that the mean WBC concentration measured on day 28 of niliotinib treatment was similar to that measured post-SCT-pre-nilotinib and remained stable during follow up is therefore of importance. It appears that nilotinib did not impair patients' leukopoiesis or result in prolonged leukopenia after SCT.

It is well established that balanced recovery of CD4 and CD8 T-cell subsets is needed to control alloimmunity and establish immune tolerance [53, 54]. We demonstrate that CD8 T cells quickly reached normal levels and continued to increase until day 180 after transplant. CD4 T-cells remained below normal until 180–270 days follow-up period. Recovery of CD4 T-cells was somewhat faster in comparison to previous publications, which had demonstrated CD4 T-cells below normal throughout one year after SCT [55, 56]. Similar to T-cells, B-cell counts were shown to be deeply depressed immediately after transplant, reaching normal levels only by nine months after allogeneic SCT [57]. In our cohort, levels of CD20^pos^ B cells were attained after 180 days (approximately 6 months). Again, this indicates the nilotinib does not delay immune recovery and may even facilitate it.

Overall, despite exposure to nilotinib, T-cells responded to mitogenic stimulation and, even more importantly, no functional deficit was observed. There was, however, a decline in T-cell mitogenic response between day 90 to day 180, which might be due the disappearance of graft T-cells that are reported to be present in the recipient blood up to approximately 3 months after transplantation [28, 50, 58, 59]. These cells provide early post-transplant protection against infection, and are reported to be replaced between day 180 to day 335 by the newly reconstituted stem-cell originated T-cells. Our results differ from other studies that showed nilotinib treatment inhibit PHA and IL-2-induced proliferation and activation markers (CD69 and CD25) on CD8+ T lymphocytes in a dose-dependent manner [29, 32]. These differences may be due to our having analyzed T-cell function after relatively prolonged treatment with nilotinib, enabling T-cells to better adapt to and tolerate the drug. Furthermore, measuring T-cells function at several defined time-points during the treatment with nilotinib allowed us to monitor the different tempos of T-cell reconstitution.

Effective reconstitution of T-cells following SCT requires a functional thymus, particularly in the case of CD4^+^ T cells [60]. With regard the thymic activity, in our study both TREC levels and surface membrane expression of different TCR Vβ families were detected at the end of follow-up in patients who received SCT and nilotinib treatment. Notably, despite being heavily immunosuppressed from previous procedures and leukemia, patients were able to reconstitute their thymic activity. TREC quantification is a suitable and practicable method to monitor thymic output post-SCT in adult patients [61], and has been shown to be an robust assay for determining immune reconstitution and for the prediction of morbidity and mortality after SCT [62]. The process of T-cell neogenesis after SCT becomes evident by 6 months, and normal levels of adult thymic functions are restored at 12 months [63]. In parallel, the TCR repertoire is considerably abnormal during the first year after transplantation, but normalizes by the time of full immune reconstitution, as was seen in our patients.

NK cells sub-population is one of the pillars of innate immunity. Our findings show that NK cells were reconstituted by one month post-transplant. These early reconstituting cells are characterized as donor-derived NK cells that effectively lyse recipient leukemia cells *in-vitro* [59], and are thus very important in preventing disease relapse. We evaluated NK cell function before and during nilotinib administration (up to 335 days) by their cytotoxic activity against K562 cell line in different E:T ratios. We demonstrated that the percent of target-cell-specific death by patients' NK cells was stable at post-SCT-pre-nilotinib through day 270 of nilotinib administration. A small but statistically insignificant increment in cell-specific killing was found at day 335. Since it is known that NK cells reconstitute by the first month following allo-HSCT, evaluation of patients' NK activity pre-nilotinib administration may serve as a valuable control for assessing the effect of nilotinib therapy on NK function. Overall, our results indicate that nilotinib did not attenuate natural killer cytotoxic capabilities. These results align with those presented by Salih *et al,* which showed no effect of nilotinib administration on cytotoxic activity of NK cells obtained from CML patients, although their cytokine production was significantly reduced [30].

Taken together, our results indicate that use of nilotinib as maintenance therapy following allogeneic SCT in CML and Philadelphia positive ALL does not jeopardize immune reconstitution; WBC counts, lymphocyte subpopulation, and *ex-vivo immune* functions recovered as expected, concomitant with the nilotinib exposure. Consequently, we suggest that all patients with Ph-positive ALL and advanced stage CML are candidates for the post-transplant use of TKIs to reduce the risk of relapse. The issue of TKI treatment after allo-SCT remains a relatively poorly explored area of investigation with many open questions requiring further research. Rigorous monitoring of MRD allows the identification of the patients who will benefit most from TKI treatment after allo-SCT. A preemptive strategy should be applied only if adequate monitoring of BCR-ABL transcripts is available with the use of real-time quantitative PCR [64]. Patients who cannot be rigorously monitored for the MRD status should be treated prophylactically. Even in cases of strict monitoring, the sensitivity of the methods used for MRD detection varies, with real-time quantitative PCR still insufficiently standardized [65].

Future prospective comparative studies should explore immunological status during treatment with different TKIs after allo-SCT: should TKIs be used as a mean of relapse prophylaxis, or as preemptive therapy triggered by the detection of MRD; what kind of TKI should be used; which TKI is better tolerated in the early period after allo-SCT; and what is the optimal treatment duration with a TKI.

## MATERIALS AND METHODS

### Patient characteristics

The complete eligibility criteria and the clinical characteristics of the cohorts were previously described by Shimoni *et al* [23]. Of the original 16 patients who participated in the clinical study, full immunological evaluation was available on 12 patients receiving nilotinib for at least 90 days following allo-SCT (Table 3). Three of the 12 patients also received nilotinib prior to SCT. Patients were included in the study regardless of the number or types of previous therapies. The schedule of post allo-SCT nilotinib administration is described in detail by Shimoni et al. [23]. In the 12 patients included in the current analysis, nilotinib was started at a median of 55 days (range 33–200) after allo-SCT. Nine patients received nilotinib at a dose of 200 mg × 2/day and three patients received nilotinib at a dose of 300 mg × 2/day, respectively. Due to increased toxicity, none of the patients received nilotinib at the highest dose of 400 mg × 2/day [23]. All patients received cyclosporine as part of post-transplant GVHD prophylaxis. At least 50% of the patients received steroids during the study period. The dose and treatment duration were different and the subgroups were too small to stratify patients for immunological assessment.

**Table 3:** Patients' characteristics and outcomes

N	Age	Gender	DS/Indication for SCT	Previous treatment	Status before SCT	Nilotinib before SCT	Donor's type	Conditioning	NILO doses after SCT	OS (month)	GVHD		DFS (months)	Mortality	
											aGVHD	cGVHD		NRM	Relapse
1	24	M	ALL Ph+	GMALL+IMA	Morphological CR;RT-PCR 1.8%	No	Haplo	ARA-C+ BU+ CY (MA)	200 mg × 2/d	35.5	Yes GI, grade II	Yes extensive skin	23.3	No	Yes
2	43	M	CML BC	Induction 7+3 + IMA	CHR; CP2 FISH t9;22 75%	No	MUD	FLUBU4 (MA)	200 mg × 2/d	Alive CR 90	Yes GI, grade II	Yes extensive skin	90	No	No
3	39	M	CML AP	HU, IMA	CP	Yes	SIB	BUCY (MA)	200 mg × 2/d	Alive CR 21^*^	Yes Skin grade II	Yes extensive skin	21^*^	No	No
4	25	M	CML BC	Induction 7+3 + IMA	MMR	No	SIB	CYTBI (MA)	200 mg × 2/d	Alive CR 89.4	No	Yes extensive skin, eyes, pericard	89.4	No	No
5	37	M	ALL Ph+	GMALL+IMA	Morphological CR; FISH neg	No	CB	FLU+BU+ Thiotepa (MA)	200 mg × 2/d	34.1	Yes skin grade I	Yes limited	34.1	YesSepsis	No
6	33	F	CML BC	HD ARA-C + Mitoxantrone+ IMA	MMR	Yes	SIB	BUCY (MA)	200 mg × 2/d	15.8	No	Yes limited	15.8	Yes TTP, ARF, Brain bleeding	No
7	39	F	ALL Ph+	GMALL+IMA	Morphological CR;RT-PCR 0.1%	No	SIB	CYTBI (MA)	200 mg × 2/d	Alive CR 97.1	Yes skin grade I	Yes extensive skin	97.1	No	No
8	37	M	CML. Lymphatic BC	GMALL+IMA	CHR;RT-PCR 128%	No	MUD	CYTBI (MA)	200 mg × 2/d	42.9	Yes skin grade I	Yes limited	10	No	Yes
9	29	F	ALL Ph+	GMALL+IMA	Morphological CR; FISH neg	No	SIB	CYTBI (MA)	300 mg × 2/d	Alive CR 64.4	Yes Skin, GI grade II	Yes limited	64.4	No	No
10	57	F	CML BC	Induction 7+3 + IMA	CP; CCyR; RT-PCR 3.6%	No	MUD	FLUBU4 (MA)	300 mg × 2/d	Alive CR 78.2	No	Yes extensive	78.2	No	No
11	30	M	ALL Ph+	GMALL+IMAà Failure; HD ARA-C + Mitoxantrone	Morphological CR; FISH negRT-PCR 1.9%	No	MUD	CYTBI (MA)	300 mg × 2/d	10.2	Yes Skin, Liver grade II	Yes extensive	10.2	Yes Nocardiasis Brain bleeding	No
12	21	M	CML BC	Induction 7+3	AP CML, FISH 7%; Start Nilo 2 weeksà CHR; CCyR; RT-PCR 2%	Yes	SIB	BUCY (MA)	200 mg × 2/d	Alive CR 69.6	Yes skin grade I	Yes extensive	69.6	No	No

* Dropped from follow up after study was completed

Abbreviations: CML – chronic myeloid leukemia, BC – blast crisis, AP – accelerated phase, ALL – acute lymphoblastic leukemia, Ph+ Philadelphia positive, GMALL – German multicenter ALL protocol, IMA- Imatinib, CR- Complete remission, RT-PCR – Real time polymerase chain reaction, ARA-C – cytosine arabinoside, HD – high dose, BU - Busulfan, CY - Cytoxan, HU - Hydroxyurea, TBI – Total body irradiation, MA - myeloablative, NILO - Nilotinib, OS – Overall survival, DFS – Disease free survival, NRM – Non-relapse mortality, GVHD- Graft versus host disease, CR – Complete remission, F - Female, M - Male, CHR – Complete hematologic response, FISH – Fluorescence in situ hybridization, MMR – Major molecular response, CMR – Complete molecular response, CCyR – Complete cytogenetic response, MUD – Match unrelated donor

### Sampling, cell source and cell culture

**Figure 4:**
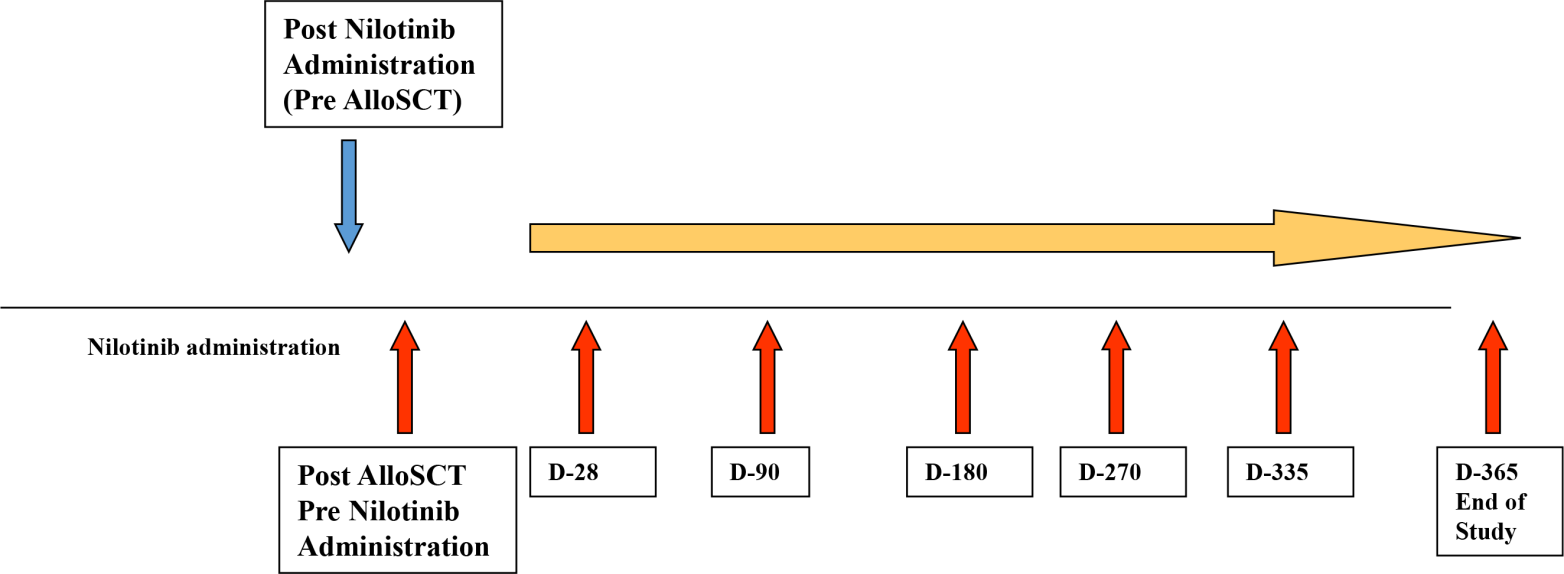
Study design of immunological assessment following SCT and nilotinib administration Patients' samples were collected at different time post nilotinib administration (red arrows). Samples obtained from patients post SCT, before nilotinib administration served as a specific control.

All 12 patients received nilotinib for at least 90 days following allo-SCT. Up to 60 mL of whole blood were collected from consenting patients at different time points, as illustrated in Figure 4 (pre-nilotib post-allo-SCT, and within 28, 90, 180, 270, 335 or 365 days of nilotinib administration). The first time-point post allo-SCT pre-nilotinib treatment served as an internal control for each patient.

Peripheral Blood Mononuclear cells (PBMNCs) were obtained from whole blood samples by density gradient (1.077 g/dl) (LymphoprepTM, cat. #1114547, Fresenius Kabi Norge AS, Norway). Patients' Natural Killer cells (NK) were isolated immediately using Miltenyi Biotec NK Cell Isolation Kit (Cat # 130-092-657) in accordance with manufacturer instructions. After isolation, cells were resuspended in 1640 RPMI medium supplemented with 10% FCS, 2 mM/ml L-glutamin, 100 U/ml Penicillin-Streptomycin Solution, (all produced by Biological Industries, Beit Haemek, Israel) and 100 IU/ml of IL-2 (Peprotech, Israel). NK cells were incubated overnight in a humidified CO_2_ incubator at 37°C before being used as effector cells in the killing activity tests performed. A K562 cell-line was used as target cells for killing activity assays. Cells were cultured in 1640 RPMI medium supplemented with 10% FCS, 2 mM/ml L-glutamin, 100 U/ml Penicillin-Streptomycin Solution, (all produced by Biological Industries, Beit Haemek, Israel). Eighteen hours prior to the killing assay, cells were collected and stained with 10 nM CFSE (5–6 Carboxyfluorescein Succinimidyl Ester) (Ebioscience).

### Immuno-phenotyping of T cells subpopulations

The immune phenotyping was performed using a multi-color flow cytometry (FACS) with the following antibodies: CD3-FITC (Becman-coulter) for total T-lymphocytes, CD4-PE (BC), CD8-PE (BC), CD16-FITC (BC), CD20-PE (IQP) for total B-lymphocytes and CD56-PE (BC) for natural killer cells, all gated on CD45 bright and SSC low. The following staining panels were performed: CD3-CD4; CD3-CD8 and CD16-CD56-CD3. A total of 1–5 × 10^5^ cells were analyzed with FACS-Canto II (BD Bioscience). The data analysis was performed by FACS-DIVA software (version II, BD bioscience).

### T-cell mitogenic response

To evaluate patients' T-cell mitogenic response, 10^5^ PBMNCs were cultured for 72 hours in the presence of anti-CD3 antibody or phytohaemagglutinine (PHA) at two different doses: 5 μg and 25 μg, respectively. Cell proliferation was assessed by measuring DNA content using a single pulse of 1 μCi [3H] TdR (Rotem Industries, Inc., Israel), eighteen hours before measuring 3H content by β-counter. Stimulation Index (SI) was calculated using the following equation:

$$\text{SI}=\frac{\text{CPM}(\text{stimulated})}{\text{CPM}(\text{unstimulated}).}$$

### Cytotoxic activity of NK cells

Target and effector cells were collected after overnight incubation, washed once by centrifugation at 1250 rpm for 10 minutes, resuspended in 1 ml complete medium and counted with hemocytometer. A killing assay was performed by co-culturing constant numbers (25,000) of CFSE-labeled K562 and NK cells in 0.5:1, 1:1, 5:1 Effector:Target (E:T) ratios in 96 wells plate (v bottom). Co-cultures were incubated for 4 hours in humidified 5% CO_2_ incubator at 37°C. Target and effector cells were incubated separately to measure basal cell death. Four hours after co-culture, cells were collected, washed with PBS-1% BSA, and stained with 25 μL/mL 7-amino actinomycin D (7-AAD, E-Biosciences) for 10 minutes at room temperature (R/T). Two-color flow cytometry acquisitions were performed immediately (no longer than 1 hour post staining.) Dead cells were measured by double CFSE^pos^ and 7AAD^pos^ cells and cytotoxic activity was expressed as % specific lysis calculated by the following formula:

$$\%\text{ specific lysis}=\frac{100\times (\%\text{ sample lysis}-\%\text{ basal lysis})}{100-\%\text{ basal lysis}.}$$

### Quantification of thymic activity

Thymic activity was evaluated by analyzing newly-derived T-cells using T-cell receptor excision circles (TREC) and the surface membrane expression of 24 different T-cell receptor (TCR) Vβ families in patient samples collected before and at the last nilotinib treatment time-point. TREC copy numbers were determined using quantitative real-time PCR (qRQ-PCR). PCR reactions were performed as previously described using 0.5 μg genomic DNA (gDNA) extracted from the patients' PBMCs as template. qRQ-PCR was carried out using an ABI PRISM 7900 Sequence Detector System (Applied Biosystems). A standard curve was constructed by using serial dilutions containing 10^3^ to 10^6^ copies of a known TREC plasmid. Patient and control samples were tested in triplicate, and the number of TRECs in each sample was calculated by comparing the obtained cycle threshold (Ct) value of the sample to the standard curve using an absolute quantification algorithm. Surface expression of individual TCR-Vβ families was analyzed using flow cytometry and a set of Vβ-specific fluorochrome-labeled monoclonal antibodies (Becton-Dickinson, Calibur) as described [48]. Normal control values were obtained from the IOTest Beta Mark-Quick Reference Card (Beckman Coulter).

### Statistical analysis

Longitudinal changes in continuous variables were assessed by paired *t*-test. *P*-values of 0.05 were considered statistically significant.

### Informed consent

Patients gave written informed consent, and the study was approved by the institutional review board. The study was registered at ClinicalTrials.gov as NCT00750659.
